# Genomic Signatures in HPV-Associated Tumors

**DOI:** 10.3390/v13101998

**Published:** 2021-10-05

**Authors:** Suleman S. Hussain, Devon Lundine, Jonathan E. Leeman, Daniel S. Higginson

**Affiliations:** 1 Department of Radiation Oncology, Memorial Sloan Kettering Cancer Center, New York, NY 10065, USA; hussains@mskcc.org (S.S.H.); devonlundine@gmail.com (D.L.); 2 Department of Radiation Oncology, Dana-Farber Cancer Institute, Harvard Medical School, Boston, MA 02189, USA; jonathane_leeman@dfci.harvard.edu

**Keywords:** E6 E7 oncoproteins, human papillomavirus, DNA repair, alternative end-joining

## Abstract

Papillomaviruses dysregulate the G1/S cell cycle transition in order to promote DNA synthesis in S phase, which is a requirement for viral replication. The human papillomaviruses (HPV) E6 and E7 oncoproteins mediate degradation of the cell cycle regulators p53 and Rb, which are two of the most universally disrupted tumor-suppressor genes in all of cancer. The G1/S checkpoint is activated in normal cells to allow sufficient time for DNA repair in G1 before proceeding to replicate DNA and risk propagating unrepaired errors. The TP53 pathway suppresses a variety of such errors, including translocation, copy number alterations, and aneuploidy, which are thus found in HPV-associated tumors similarly to HPV-negative tumors with other mechanisms of TP53 disruption. However, E6 and E7 maintain a variety of other virus–host interactions that directly disrupt a growing list of other DNA repair and chromatin remodeling factors, implying HPV-specific repair deficiencies. In addition, HPV-associated squamous cell carcinomas tumors clinically respond differently to DNA damaging agents compared to their HPV negative counterparts. The focus of this review is to integrate three categories of observations: (1) pre-clinical understanding as to the effect of HPV on DNA repair, (2) genomic signatures of DNA repair in HPV-associated tumor genomes, and (3) clinical responses of HPV-associated tumors to DNA damaging agents. The goals are to try to explain why HPV-associated tumors respond so well to DNA damaging agents, identify missing pieces, and suggest clinical strategies could be used to further improve treatment of these cancers.

## 1. Introduction

The high-risk human papillomaviruses (HR-HPV) infect the basal layer of epithelium in the anogenital region and the oropharynx. While most infections are cleared, in some individuals, HR-HPV infections and the introduction of oncoproteins E6 and E7 is the founding carcinogenic event for the development of squamous cell carcinomas arising from these anatomic locations. While sufficient for the immortalization of keratinocytes, E6 and E7 are insufficient for transformation into malignancy, and the acquisition of additional driver alterations is required. In murine models, the expression of HPV18 E6/E7 alone does not induce malignancy, which also requires an additional oncogene (ras or fos) [1]. In human HPV-associated cervical, oropharyngeal, and anal cancers, these additional drivers include copy number alterations and driver gene mutations affecting tumor suppressor genes or oncogenes [2,3,4]. In addition, numerous passenger alterations in non-driver elements are also acquired. Inferences about how these driver and passenger alterations are formed can be gleaned by evaluating patterns or “signatures” of mutations and structural rearrangements [5,6]. A signature is a reflection of an underlying genomic insult (e.g., a pyrimidine dimer from UV light) and how the early cancer cell repairs that insult. Since the E6/E7 oncoprotein expression is a first step in carcinogenesis, the impact of these oncoproteins on fundamental processes such as DNA repair and genomic stability can be discerned through measurement of the genomic signatures acquired in cancer genomes on the course from HPV infection to clinical tumor detection. Two of the most important features of the oncoproteins E6 and E7 include the inactivation of p53 and Rb; however, virtually all cancers also have acquired mechanisms of inactivation of these targets as well. For instance, nearly all HPV− head and neck squamous cell carcinomas have acquired TP53 mutations and loss of CDKN2A [2], providing an alternative means of bypassing the Rb-mediated restriction point and the G1/S checkpoint enforced by p53. Thus, evaluating genomic differences between HPV+ and HPV− cancer genomes can help distinguish between E6/E7 effects on DNA repair and genomic stability other than simply inactivation/loss of TP53. These comparisons between HPV+ vs. HPV− tumor genomes comparison can best be done between head and neck squamous cell carcinomas as the overwhelming majority of cervical cancers and most anal cancers are HPV+.

## 2. Radiosensitivity Differences between HPV+ and HPV− Tumors

Locoregionally advanced head and neck squamous cell carcinomas are often best managed with concurrent chemoradiation, consisting of 70 Gy in 2 Gy fractions with concurrent cisplatin either weekly or in three bolus doses over the course of radiotherapy [7,8]. The value of the cisplatin in addition to radiotherapy was demonstrated in multiple randomized trials, [7,8] including two recent dedicated HPV-associated oropharyngeal SCC trials [9,10]. Concurrent cisplatin is similarly supported by phase III trials in squamous cell carcinomas of the cervix [11,12,13], and in the case of anal canal squamous cell carcinoma, the crosslinking agent mitomycin C substituted for a platinum salt also improves outcomes [14,15]. However, the primary curative modality is the radiotherapy since chemotherapy alone is non-curative, but the addition of concurrent cisplatin improves the 10-year locoregional cure rate in HNSCC from 54 to 71% compared to radiation alone [8].

In 2008, a landmark analysis of a prospective Eastern Cooperative Oncology Group (ECOG) trial of concurrent chemoradiation for head and neck cancers established superior outcomes for HPV-associated squamous cell carcinomas in terms of local control following radiotherapy as well as overall survival [16]. The superior radiosensitivity of HPV-associated HNSCC compared to HPV negative HNSCC has been confirmed in multiple other trials [17,18] and is also observed in SCC of the cervix [19,20], vulva [21,22,23], and anal canal [24,25,26], suggesting a consistent biology. Using HPV association as a biomarker, multiple phase II trials have now supported radiation dose reductions to 60 Gy [27,28], 54 Gy [29,30], or even 30 Gy [31], which is not possible in HPV-negative HNSCC. However, recent studies have shown that patients with integrated HPV genomes (a subset of 20% of all HPV-positive patients) have poor clinical outcomes compared to patients with episomal HPV; thus, we might need to further stratify patients before selecting them for de-escalation therapy [32,33].

Pre-clinical models of HPV-associated HNSCC are few, but multiple groups have replicated the demonstrated increased radiation sensitivity in collections of HPV+ HNSCC cell lines compared to HPV− HNSCC cell lines using gold standard clonogenic survival assays [34,35]. In patient-derived xenograft models, HPV+ HSNCC similarly are generally more radiosensitive compared to HPV− models [36]. These comparisons are made between collections of genetically heterogeneous cell lines, but isogenic comparisons of cell lines with and without E6 and E7 expressed also demonstrated HPV oncogene-mediated radiation sensitivity [37]. To compare radiation responses in implanted xenografts, Brezar et al. constructed an isogenic pair of the commonly used HPV negative HNSCC cell line FaDu and an FaDu line expressing E6/E7 [38]. Following 10 Gy of radiation, the E6/E7 FaDu xenograft exhibited a five-fold growth delay relative to the parental FaDu xenograft [38].

## 3. Proposed Mechanisms for Increased Radiosensitivity in HPV+ Tumors

The cell-intrinsic radiosensitivity found in clonogenic survival assays has been linked to a DNA double-strand break repair defect. Again using a comparison of cell line collections in HPV+ and HPV− categories, the resolution of γH2AX foci after radiation is delayed in HPV+ cell lines, suggesting a defect in DSBR (double strand break repair) [34,35]. Isogenic comparisons also confirm a defect, and when E6/E7 or E7 alone are expressed in immortalized keratinocytes or transgenetically introduced into skin keratinocytes in a murine model, the resolution of γH2AX is delayed [37]. Finally, when E6/E7 are introduced into a primary model of oral cancer, γH2AX is increased [39].

**Figure 1:**
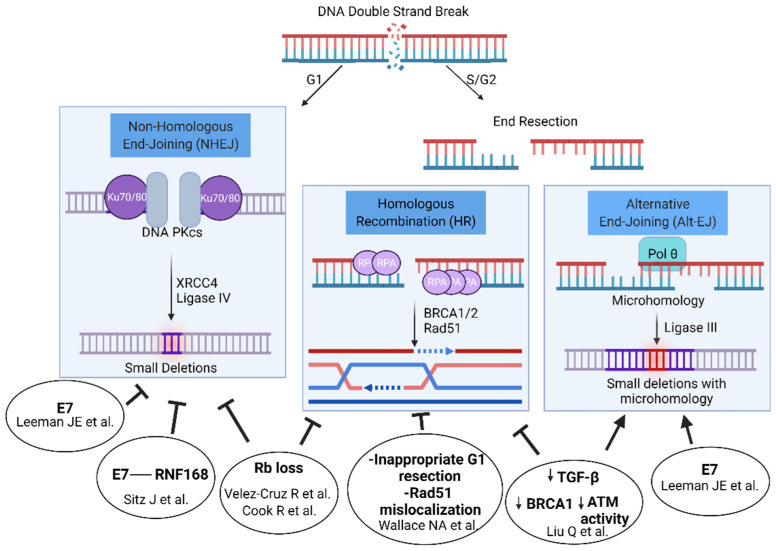
Double-strand break repair pathways and proposed mechanisms of HR-HPV mediated effects on pathway usage. Figure created with Biorender.com.

Both E6 or E7 individually and joint E6/E7 expression have been linked to DSBR defects and multiple DSBR pathways are proposed to be abrogated, including both homologous recombination [40,41] and nonhomologous end-joining [42,43]. Thus, there may not be one single explanatory mechanism but rather multiple (see Figure 1). Both E6 and E7 increased persistent γH2AX signal after radiation when expressed individually in the HPV-negative FaDu cell line [44]. However, primarily E7 delayed resolution of γH2AX in immortalized keratinocytes and transgenic models of E6 and E7 expression in skin [37] and only E7 or E6/E7 led to a DSB defect in an isogenic U2OS cell line model [42].

Following a double-strand break created by irradiation, an estimated 70% of breaks are repaired through nonhomologous end joining, a rapidly completed pathway consisting of end recognition by the XRCC5/6 heterodimer and DNA-PK, which forms a synaptic complex across the break site. Compatible ends can be directly ligated through Ligase IV, or limited end processing can occur to remove oxidatively damaged residues before subsequent ligation. In S and G2 cells, CTIP and the MRN complex can initiate end-resection, leaving a single strand 3′ prime end, which can invade a sister chromatid strand, find homology, and complete homologous recombination. A less well-characterized pathway, alternative end joining (Alt-EJ), also makes use of the exposed single stranded 3′ end from each side of the break. Small stretches of homology between opposite strands of the break, termed ‘microhomology’, can be annealed, initiating repair through polymerization and the clipping of non-complementary tails.

HPV E6/E7 expression has been linked to homologous recombination deficiency. Liu et al. have described an abrogation of TGF-β signaling in HPV+ HNSCCs and a subsequent de-repression of miR-182, which in turn inhibits BRCA1 and FOXO3 expression, thereby inhibiting homologous recombination directly through BRCA1 and indirectly through FOXO3 effects on ATM activity [41]. Inhibition of TGF-β signaling suppressed homologous recombination, increasing alternative end joining. Furthermore, when the expression of TGF-β genes are analyzed pan-cancer, this TGF-β signature is inversely correlated with expression of alt-EJ genes. An impact on ATM activation was also seen through E6-mediated degradation of TIP60, which is an acetyltransferase involved in the activation of ATM [45]. E7 also activates STAT5, causing increased phosphorylation of both ATM and ATR [46,47]. Furthermore, this activation of the JAK/STAT pathway helps HPV evade the immune system, and thus, targeting the JAK/STAT pathway could be another strategy to treat HPV-associated tumors [48]. Wallace et al. similarly demonstrated the inhibition of homologous recombination when E6 is expressed in a U2OS cell background, a 50% reduction in homologous recombination through an inappropriate initiation of homologous recombination in G1, and mislocalization of Rad51 away from DNA damage foci [40]. Additionally, the E7 oncoprotein induces the overexpression of the p16ink4a (p16) cell cycle inhibitor protein, and p16 may play indirect roles in homologous recombination [49]. p16 inhibits CCND1 expression, which in addition to its role in cell cycle progression also is proposed to have a direct role in homologous recombination [50]. p16 overexpression also decreases TRIP12 expression, an E3 ubiquitin ligase, and subsequently the overaccumulation of RNF168 [51]. Finally, the Rb protein itself (which is degraded by E7) has been demonstrated to promote HR through recruitment of the BRG1 ATPase, increasing end resection possibly through reducing the density of nucleosomes surrounding a DSB [52]. Thus, a number of different proposed mechanisms link E6/E7 oncoproteins to an HR defect.

In contrast, when comparing panels of HPV+ and HPV− cell lines that did exhibit separable radiation sensitivities in the hands of multiple groups [34,35], no such difference can be observed in cisplatin sensitivity [53]. Cisplatin mediates cytotoxicity through interstrand crosslinks, which require an active HR pathway to resolve. Response to platinum is a hallmark feature of HR-deficient cancers such as germline mutated BRCA1/2 breast and ovarian cancers.

The E6/E7 oncoproteins have also been proposed to influence repair through end joining. Using three different methods, our group measured the impact of E7 on DSBR and found an E7-mediated suppression of NHEJ, with a concomitant increase in Alt-EJ and HR, similar to the phenotype of NHEJ-inhibiting drugs [42,54]. The mechanism may be explained by the effect of E7 on viral replication centers, which are extrachromosomal DNA structures formed during the life cycle of the virus. A number of DNA repair proteins are recruited to viral replication centers, including ATM, the MRN complex, BRCA1, and 53BP1 [55,56,57,58]. Sitz et al. found that E7 binds RNF168, and the interaction is required for viral replication [43]. RNF168 ubiquitinates the histone protein H2A [59], which is one of two important histone markers that recruit 53BP1 to double-strand breaks. The 53BP1/RIF1/shieldin complex axis serves as the primary mechanism to protect the ends of a double-strand break from resection, thus promoting nonhomologous end joining over homologous recombination [60,61,62,63]. E7 expression substantially reduced 53BP1 foci in response to radiation (a marker of NHEJ) and promoted homologous recombination through a reporter cassette assay [43]. Another possible mechanism lies in the Rb protein itself. Rb is also reported to mediate the recruitment of XRCC5/6, key NHEJ effectors, to double-strand breaks, and Rb knockdown reduces NHEJ efficiency by cassette reporters [64].

While many studies have focused on how E6 and E7 modulate DSBR, viral replication itself requires DDR activation [56], including activation of ATM and ATR signaling pathways [46,57,65,66]. Homologous recombination proteins are also required for productive replication [65], and various DSBR factors are recruited to viral replicating centers, including ATM, BRCA1, RAD51, NBS1, ATM, WRN, and TOPBP1 [67,68,69,70]. The E1 and E2 proteins induce the DNA damage response as seen by increased γH2AX foci and DSBs measured by COMET assay in S and G2 phases [67,71]. In addition, the E2 protein can bind directly to several proteins involved in the DNA damage response and modulate their function, including TopBP1 [68,72]. These observations imply that viral replication may involve DNA structure complexity that requires DNA repair to proceed. A plurality of HPV-associated cancers maintains episomal DNA copies of HPV genomes, which could be replication-competent [2,3,32,33], and E2, E4, and E5 can remain expressed in some HPV+ cancers [73]. How replication competence affects overall DSBR in HPV+ tumors is unclear, and a worthwhile comparison would be episomal only vs. integrated HPV+ cancers in terms of sensitivity to DNA damaging agents and DSBR pathway utilization.

In addition to the DNA repair related, cell-intrinsic radiosensitivity mechanisms, other possibilities include incomplete TP53 inactivation [34], expression of radiosensitizing E6 isoforms [74], or prolonged G2/M arrest [35]; there are also cell-extrinsic proposed mechanisms involving the tumor microenvironment, such as decreased tumor hypoxia and improved immunoreactivity, which are nicely reviewed elsewhere [39,75,76].

## 4. Genomics Signatures in HPV Cancers

A complementary approach to cell-based analyses of DSBR is to analyze cancer genomes to measure characteristics DNA repair scars (“signatures”) that can reflect the underlying DNA repair capacity of the tumor. Examples include cancers defective in mismatch repair exhibiting microsatellite instability in the genome and cancers defective in homologous recombination exhibiting a variety of associated genomic alterations. What can the underlying genomics of HPV+ cancers teach us about DNA repair in these cancers?

First, we know that the expression of E6/E7 into cells can cause genomic instability, which is reflected by rearrangements, translocations, and amplifications [77,78], and also changes in ploidy [79]. Of course, all these measures of genomic and chromosomal instability are associated with TP53 loss, which is also mediated through E6. HPV− tumors exhibit near universal TP53 genetic inactivation. When HPV+ and HPV− HNSCC genomes are directly compared, HPV+ HNSCC actually exhibit fewer copy number alterations compared HPV− HNSCC genomes (median 113 vs. 136, *p* = 0.026) and a higher percentage of HPV+ HNSCC genomes are classified as “M” type, driven by more mutations in cancer drivers than copy number changes (58% vs. 27%) [2]. Thus, it is not clear from raw measures that HPV+ genomes exhibit genomic instability over and above that of other tumors, such as HPV− tumors with TP53 loss (see Table 1).

Second, the overall frequency of mutations does not differ between HPV+ and HPV− HNSCC genomes. However, the mode of acquisition of single base substitutions does differ substantially. HPV− genomes are dominated by C>A substitutions induced by smoking, whereas HPV+ genomes exhibit dominance of the APOBEC signature involving C>T and C>G mutations in TpCpN trinucleotides. The APOBEC cytidine deaminases mediate anti-viral activity through deamination of T to U in single-stranded viral DNA. As a harmful by-product, single-stranded DNA occurring in the genome during replication can similarly be affected by APOBEC enzymes, inducing base substitutions.

Third, when focusing on genomic signatures present in cancer deficient in homologous recombination, HPV+ tumors do not exhibit SBS3, the single base substitution pattern found in BRCA1/2 biallelic mutated cancers defective in HR [80], and if anything, the SBS3 contribution is slightly higher in HPV-negative HNSCCs [42,80,81]. Furthermore, BRCA1/2 mutated cancers exhibit an increase in large-scale state transition (LST) score, which is reflective of large rearrangements [82], but HPV+ HNSCC genomes do not exhibit this hallmark, either [42].

What is different between HPV+ and HPV− tumors is the frequency of deletions associated with microhomology-based repair used in alternative end joining [42]. Those HPV+ tumors with the highest levels of E7 expression exhibited a still higher percentage of Alt-EJ like scars. Finally, HPV+ tumors with high levels with ALT-EJ genomic scars exhibited improved disease-free survival following radiation (3-year DFS 60.1 vs. 41.2%, *p* = 0.04) [42]. Subsequently a prevalence of Alt-EJ genomic scars (termed ID6 in this case) without SBS3 was similarly demonstrated in cervical cancer whole genomes [83]. To reconcile the absence of HR-associated base substitution (SBS3) and large-scale state transition (LST) signatures and the presence of Alt-EJ deletion signatures, one explanation could be that the Alt-EJ signatures are reflective of defective NHEJ. Alternative end joining was first discovered in V(D)J junctions within lymphocytes deficient in NHEJ [84,85] and thus is a backup to canonical NHEJ, just as it is a backup to homologous recombination. Finally, the Alt-EJ signature and its relation to clinical outcomes in HPV+ HNSCCs was further confirmed in a prospective study [86].

## 5. Clinical Responses to Platinum in HPV+ Cancer

Another means of assessing real-world homologous recombination deficiency is to compare the clinical response rates of recurrent and metastatic HPV+ tumors to platinum salts and PARP inhibitors to those of HPV− tumors. Platinum and PARP inhibitor sensitivity are largely determined by HR capacity. For instance, the overall response rates to platinum in germline bi-allelic mutant BRCA1/2 cancers range from 65 to 95% [87,88,89,90,91,92]. In germline BRCA1/2 pancreas cancers, the ORR to cisplatin/5-FU is 65% [87,88]. In prostate cancer, these BRCA1/2 cases respond to carboplatin/taxol at a rate of 75% [89]. In breast cancer, not typically treated with platinum salts, 68% of biallelic mutant BRCA1/2 cases respond to cisplatin/gemcitabine [90]. Finally, in ovarian cancer, germline BRCA1/2 cases respond in 87–96% of cases in prospective trials [91,92]. In HPV+ HNSCCs, the clinical response rate was recently measured in the standard of care cisplatin/5-FU arm of the practice changing EXTREME trial. For all HNSCCs, the ORR in HPV+ cases was only 22%, which is marginally different that the 17% response rate observed in HPV− cases. When narrowed to only the oropharyngeal HPV+ cases, the ORR was 24% compared to 21% in HPV− disease [93]. Thus, the response rates of HPV+ tumors to platinum salts are not similar to true HR-deficient cancers, although a smaller, relative HR defect cannot be excluded.

## 6. Summary and Discussions

The radiosensitivity of HPV+ HNSCC, anal and vulvar cancers is a clinically important topic, as few other biomarkers of radiation sensitivity exist. In pre-clinical models, there is a considerable body of work supporting both HR and NHEJ deficiency induced by E6/E7. HPV+ tumor genomes demonstrate an absence of canonical HR deficiency signatures, such as SBS3 and LST, but they do exhibit increased Alt-EJ scars relative to their HPV− counterparts, possibly supporting increased Alt-EJ in response to a partial NHEJ deficiency. Finally, available clinical data strongly support a role for cisplatin in concurrent chemoradiotherapy, but as a stand-alone agent, it does not result in the kind of response rates common in true HR-deficient cancers. Opportunities for improvement in this field include additional prospective validation of Alt-EJ signatures and clinical investigation of DNA damage response inhibitors that can best take advantage of HPV-associated DSBR defects.

## 7. Patents

JEL and DSH are listed inventors on a patent filed by Memorial Sloan Kettering Cancer Center regarding the use of alternative end-joining genomic signatures to predict radiation sensitivity in connection with prior work [42]. There are no licenses or royalties.

## Figures and Tables

**Table 1:** Comparison of genomic characteristics and therapy response in HPV positive and negative cancers.

Characteristic	HPV+ HNSCC	HPV− HNSCC
Dysregulated tumor suppressors	Rb (E7), p53 (E6)	p16(INK4A), p53
Copy number alterations (median)	113	136
“M” class tumors	58%	27%
Somatic mutation frequency	Similar	Similar
Single base substitutions signature	APOBEC	Smoking
Alt-EJ genomic scars	Higher	Lower
